# Coupling of NIR Spectroscopy and Chemometrics for the Quantification of Dexamethasone in Pharmaceutical Formulations

**DOI:** 10.3390/ph16020309

**Published:** 2023-02-16

**Authors:** Alessandra Biancolillo, Claudia Scappaticci, Martina Foschi, Claudia Rossini, Federico Marini

**Affiliations:** 1 Department of Physical and Chemical Sciences, University of L’Aquila, Via Vetoio snc, Coppito, 67100 L’Aquila, Italy; 2 Department of Chemistry, University of Rome “La Sapienza”, Piazzale Aldo Moro 5, 00185 Rome, Italy

**Keywords:** dexamethasone, active pharmaceutical ingredients quantification, dexamethasone/lactose/starch mixtures, near infrared (NIR) spectroscopy, regression, partial least squares (PLS), sequential preprocessing through orthogonalization (SPORT), counterfeit drugs, substandard drugs, COVID-19

## Abstract

Counterfeit or substandard drugs are pharmaceutical formulations in which the active pharmaceutical ingredients (APIs) have been replaced or ingredients do not comply with the drug leaflet. With the outbreak of the COVID-19 pandemic, fraud associated with the preparation of substandard or counterfeit drugs is expected to grow, undermining health systems already weakened by the state of emergency. Analytical chemistry plays a key role in tackling this problem, and in implementing strategies that permit the recognition of uncompliant drugs. In light of this, the present work represents a feasibility study for the development of a NIR-based tool for the quantification of dexamethasone in mixtures of excipients (starch and lactose). Two different regression strategies were tested. The first, based on the coupling of NIR spectra and Partial Least Squares (PLS) provided good results (root mean square error in prediction (RMSEP) of 720 mg/kg), but the most accurate was the second, a strategy exploiting sequential preprocessing through orthogonalization (SPORT), which led (on the external set of mixtures) to an R^2^_pred_ of 0.9044, and an RMSEP of 450 mg/kg. Eventually, Variable Importance in Projection (VIP) was applied to interpret the obtained results and determine which spectral regions contribute most to the SPORT model.

## 1. Introduction

Counterfeit or substandard drugs are pharmaceutical formulations in which the active pharmaceutical ingredients (APIs) have been replaced or are present in quantities other than those declared, contain the appropriate quantity of API but other defects, including unacceptable by-products or contaminations, or have been produced without patent.

In recent years, the distribution of counterfeit medicines has grown considerably; the Pharmaceutical Security Institute (PSI) noted that, since 2018, a sharp increase in this type of fraud has been observed in the USA, Asia, and Europe [1], and the U.S. Food and Drug Administration (FDA) estimates that the percentage of counterfeit drugs in the world is 10%.

With the outbreak of the COVID-19 pandemic, this phenomenon is expected to grow, undermining health systems already weakened by the state of emergency. From this point of view, the need to have tools for the authentication of circulating medicines is even more important, in particular for all those drugs that are administered for the treatment of patients suffering from COVID-19.

Analytical chemistry plays a key role in tackling the problem of counterfeit pharmaceutical formulations, as it allows the implementation of strategies that permit the recognition of uncompliant drugs. Over the years, various analytical techniques have been refined for this purpose; in particular, liquid chromatography strategies are commonly exploited to achieve this goal [2,3,4,5,6,7,8,9,10,11,12,13]. Although efficient, they have a number of disadvantages linked to the fact that they are destructive, require complex sample pretreatment prior to the analysis, and are time-consuming. A valid alternative is represented by spectroscopic techniques, which could be non- or semi-destructive, faster, and more environmental-friendly. Several works in the literature focus on how these methodologies can be combined with chemometric approaches for the identification of counterfeit drugs; a detailed overview of this has been provided by Custers et al. [3]. The spectroscopic techniques most frequently used in pharmaceutical industries to perform quality controls are Mid- and Near-Infrared and Raman [14,15,16,17,18,19,20,21,22].

Following the current pandemic situation, the drugs in use for the treatment of patients affected by COVID-19 are of special interest, and they can be particularly subjected to fraud. Among the different pharmaceutical drugs administered to people affected by the COVID-19 virus, many of them are anti-inflammatories. In particular, in recent clinical trials, it has emerged that a specific glucocorticoid anti-inflammatory steroid, dexamethasone, is associated with a reduction in mortality in patients undergoing forced ventilation [23]. This is correlated to the capability of glucocorticoids of attuning inflammation-mediated lung injuries; and, therefore, reduce respiratory failures and deaths [23,24,25]. Consequently, given the key role dexamethasone can play in the treatment of COVID-19, it is imaginable that fraud against dexamethasone-based formulations may increase, but, at the same time, there is a clear need to preclude the spread of substandard or counterfeit medicines containing this API.

In general, dexamethasone is used for the treatment of particularly intense allergic manifestations, rheumatic, dermatological, ophthalmological diseases, adrenal endocrine disorders, respiratory system pathologies, hematological, and neoplastic diseases. It has an epimer, betamethasone, which is more expensive and has slightly different therapeutic purposes [26,27]. In the literature, some studies concerning the authentication of this drug have been conducted. This is the case of the work conducted by Rodionova and collaborators who analyzed different batches of an injectable dexamethasone-based drug [21]. Formulations were analyzed by high-performance liquid chromatography equipped with photodiode array detection-mass spectrometry (HPLC-DAD-MS), UV, and NIR. NIR spectroscopy has been used as a quality control tool for other glucocorticoid anti-inflammatory steroids, such as prednisolone [28] and cortisone acetate [29]. Through this work, the authors were able to highlight differences, particularly regarding impurity composition, between pure and counterfeit samples. Another interesting study carried out in this area is the one conducted by Arthur and collaborators, who analyzed dexamethasone and betamethasone-based drugs using reversed-phase high-performance liquid chromatography/electrospray ionization mass spectrometry (HPLC/ESI-MS) [27]. The proposed methodology appeared successful for the analysis of betamethasone or betamethasone esterification products and for the identification of a counterfeit drug.

In light of this, the present work represents a feasibility study for the development of a NIR-based tool for the quantification of dexamethasone in mixtures of excipients such as starch and lactose. In order to achieve this goal, different methodologies based on the combination of NIR and chemometric regression methods have been tested. In particular, the NIR spectra were handled using two different strategies, one based on individual-block modeling, and one based on a multi-block approach. The first strategy is based on partial least squares (PLS) [30,31] while the second exploits the sequential preprocessing through the orthogonalization (SPORT) method [32]. These two approaches were chosen because they have numerous advantages and are often accurate [33,34,35,36], particularly in this context [37,38,39].

## 2. Results

**Figure 1:**
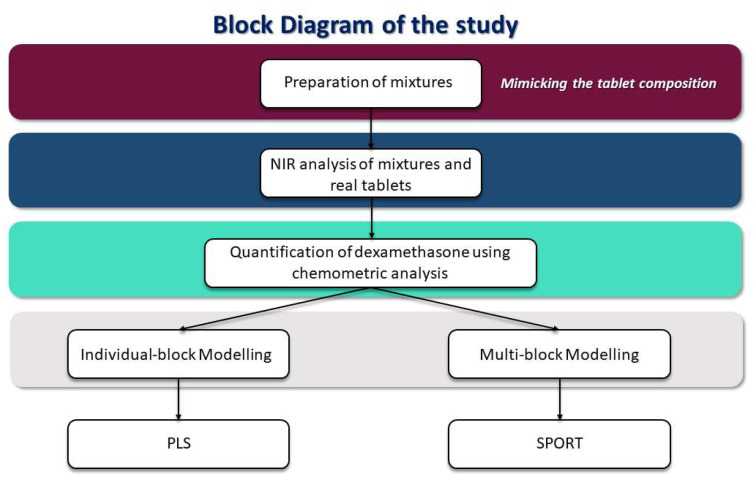
Block diagram of the study.

The present work represents a feasibility study to quantify dexamethasone in drugs using NIR spectroscopy coupled with chemometrics; the workflow of the study is shown in Figure 1. The main aim of the study is to test the possibility of developing a methodology that could help the determination of counterfeit dexamethasone-based drugs.

First, mixtures of dexamethasone together with corn starch and lactose, mimicking the possible composition of tablets, were prepared. In the different mixtures, the dexamethasone concentrations were in the range of the concentration of the API in the main formulations on the market. Mixtures were then analyzed by NIR spectroscopy and regression models for dexamethasone quantification were created. The models were developed by testing two different strategies, one based on individual block modeling, and one founded on a multi-block approach. In the first case, the Partial Least Squares (PLS) method was used to solve the regression problem, in the other, sequential preprocessing through orthogonalization (SPORT) was exploited for the same aim. Finally, the models were validated in two different ways: predicting an external set of mixtures and assessing the concentration of dexamethasone in ground tablets actually on the market (Decadron^®^, purchased in different batches in Italian municipal pharmacies).

**Figure 2:**
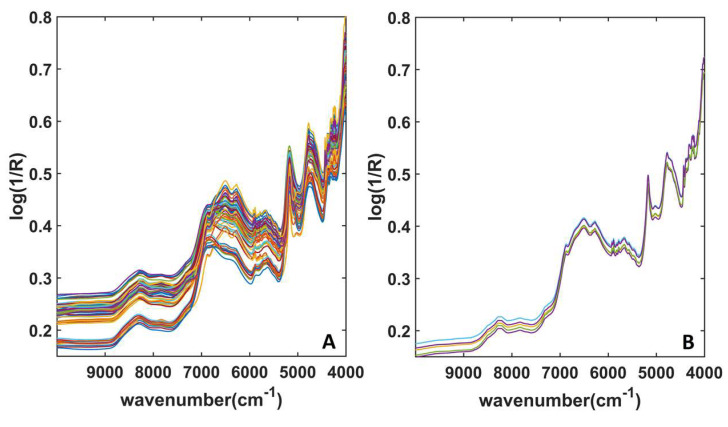
NIR Spectra acquired from (**A**) mixtures; (**B**) Decadron ground tablets.

All the collected spectra are shown in Figure 2; in particular, Figure 2A shows the signals collected on the mixtures, whereas Figure 2B displays the spectra associated with ground Decadron^®^ tablets. As can be seen, the signals do not show relevant differences. The main signals can be found around 7000 cm^−1^, i.e., associated with spectral variables ascribable to the combination of C–H stretching, and to the first overtone and the combination of O–H stretching. More details on the NIR signals can be found below in Section 2.2, where the interpretation of spectral variables through Variable Importance in Projection (VIP) analysis is discussed.

### 2.1. Strategy I (Individual-Block Modelling)

Applying strategy I (details on this strategy can be found in Section 4.3), six different Partial Least Squares (PLS) models, one for each tested pretreatment, were built. The outcome of the analysis (together with the number of latent variables (LVs) extracted) is reported in Table 1. The goodness of the models was evaluated on the basis of the coefficient of determination in cross-validation (R^2^_CV_). A value of R^2^_CV_ close to 1 can be seen as an indication of a good fit [40].

As can be observed from Table 1, models calculated on data preprocessed by the second derivative or its combination with standard normal variate (SNV) provide a good fit, shown by the relatively high coefficient of determination in cross-validation R^2^_CV_ (>0.7), attesting these as suitable pretreatments for the investigated data. The highest R^2^_CV_ (0.7785) is obtained in Model IV, i.e., when spectra are preprocessed by the second derivative (followed by mean centering (MC)); the application of Model IV for the prediction of the test set led to a root mean square error in prediction (RMSEP) of 720 mg/kg and a bias of -207 mg/kg.

### 2.2. Strategy II (Multi-Block Modelling)

As described in Section 4.3, sequential preprocessing through orthogonalization (SPORT) allows the solving of a regression problem and, simultaneously, ensemble preprocessing of data. SPORT, being a sequential multi-block method, allows for different modeling orders of the input data blocks. The order of the blocks is not expected to sensibly affect predictions, but it can lead to different outcomes from the interpretation standpoint [41,42,43]. In order to minimize these effects, SPORT models were built testing all the possible different block sequences. Models led to R**^2^_CV_** ranging between ~0.73 and ~0.84. The highest R**^2^_CV_** (0.8397) was obtained when the first modeled block was the mean-centered one (1 LV), followed by spectra preprocessed by the first derivative (7 LVs), and SNV (6 LVs). The application of this model to the test set led to an RMSEP of 450 mg/kg and a bias of -202 mg/kg.

**Figure 3:**
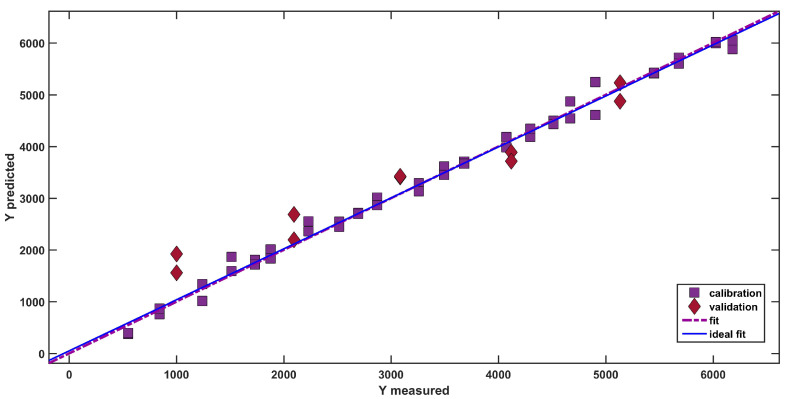
SPORT analysis: plot of predicted vs. measured dexamethasone concentration (mg/kg). The lines represent the ideal and the actual fit. Legend: Purple Squares: Calibration samples; Purple Squares: Red Diamonds: Validation samples; purple dash-dotted line: Actual Fit; Ideal Fit: Solid Blue Line.

A graphical representation of the goodness of the fit is appreciable in Figure 3, where the predicted response (dexamethasone concentration) is plotted as a function of the measured response (i.e., the known concentration of the API in the mixtures). In the figure, calibration samples are represented as purple squares and test samples are depicted as red diamonds.

As is visible from the good degree of overlap between the actual (purple dash-dotted line) and the ideal fit (solid blue line), the model is able to adequately predict the concentration of dexamethasone (R^2^_pred_ = 0.9044), providing an accurate estimation of the API concentration.

Next, Variable Importance in Projection (VIP) analysis [44] was performed to investigate which spectral variables significantly contribute to the quantification of the API. This approach allows ranking the variables according to their contribution to the model, and it provides (for each of them) an index (VIP index) proportional to their significance. By construction, the average of squared VIP values is 1; consequently, this value is often used as a threshold value to identify significant contribution, because it means that a variable presenting a VIP index higher than 1 will have an above-average influence on the model [45].

**Figure 4:**
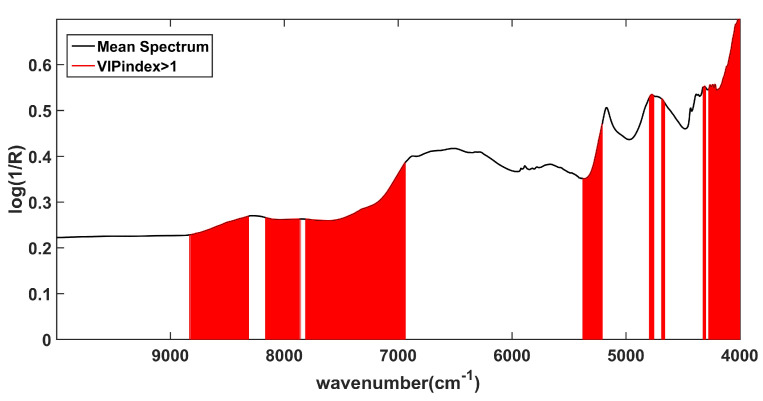
VIP analysis: identification of the variables contributing the most to the SPORT model based on the VIP indices. The black solid line represents the average spectrum and the red vertical bars represent the predictors identified as significantly contributing.

Looking at Figure 4, where the average spectrum is shown as a black solid line and variables presenting a VIP index > 1 are highlighted in red, is possible to appreciate that the spectral regions contributing the most to the model are those ascribable to the second overtone and the combination of C–H stretching (8800 cm^−1^–8300 cm^−1^, 8100 cm^−1^–7800 cm^−1^), to the first overtone and the combination of O–H stretching (7800 cm^−1^–6900 cm^−1^, 4700 cm^−1^, 4600 cm^−1^) to the combination of the C=O and O-H stretching modes and of the second overtone of C=O stretching (5290 cm^−1^).

A further SPORT model was calculated using only the variables presenting a VIP index > 1. However, it did not provide a more accurate model than the one based on all the features.

Finally, the model was used also to predict the concentration of Decadron^®^ in the analyzed ground tablets. In this case, SPORT provided satisfactory results, slightly less accurate than those obtained when validated on the external set of mixtures. Nevertheless, this is an expected phenomenon as the pharmaceutical formulation contains more excipients than those included in the mixtures. Assuming an average concentration of dexamethasone in tablets of 3333 mg/kg (in agreement with the manufacturer’s leaflet), the model achieves an RMSEP of 1020 mg/kg, and a bias of 115 mg/kg.

## 3. Discussion

The present study demonstrates the feasibility of the combination of NIR spectroscopy and SPORT for the quantification of dexamethasone in combination with lactose and starch.

The coupling of NIR and chemometric regression methods for the quantification of APIs has been already discussed in the recent literature, and the present work fits perfectly into this context. Similar accuracies have been found in other works where analogous methods have been applied to dexamethasone or other APIs. In addition to those already discussed in the Introduction, further examples can be found. For instance, the study conducted by Foo et al. [46], who applied NIR as a quality control tool for the identification and quantification of drug content in extemporaneous orodispersible films (ODFs) to lower the cost and complexity of routine analysis performed using HPLC. Calibration models were developed for the identification of ODFs containing five different drugs and for the quantification of ondansetron (OND). The qualitative model for drug identification showed 100% prediction accuracy. Two models for the prediction of OND content (in the ranges of 2 to 4 mg and 4 to 10 mg) in ODFs were calculated. These achieved RMSE values in calibration equal to 0.0856 and 0.1440, respectively, affirming the reliability of the technique. Another interesting example is the work of Cournoyer et al. [47], where a method for the quality control of intact tablets containing two different APIs, acetylsalicylic acid (ASA) and caffeine, as well as three excipients, has been developed. This study showed an uncertainty range for the quantification of ASA between 1.0 and 1.1% and an average error value of less than 5% for caffeine. In the present study, the multi-block strategy allowed the prediction of the concentration of the API in the test mixtures with an error lower than 10% for the greatest part of the validation samples, in complete agreement with these studies. In addition, VIP analysis has highlighted which variables contribute most to the quantification of dexamethasone. Due to the nature of NIR spectra, it is not possible to assign these variables to dexamethasone *per se* or to the excipients. It is possible to correlate these variables to chemical bonds, but these are present in all three constituents of the mixtures. Nevertheless, the identification of this reduced set of features can represent an excellent starting point for the development of specific devices, aimed at fast and non-destructive pharmaceutical quality controls.

## 4. Materials and Methods

### 4.1. Samples

Dexamethasone-based formulations are commercialized as tablets or liquid for injection. Oral formulations are marketed under the name Decadron or as dexamethasone tablets in Europe and the USA. In order to prepare mixtures that mimic the composition of a pharmaceutical formulation conceived for oral administration, the composition of Decadron tablets (0.5 mg) was taken as a reference. The tablets of this pharmaceutical preparation present a concentration of API corresponding to ~3333 mg/kg and contain, among the main excipients, corn starch and lactose monohydrate. Consequently, it was planned to prepare mixtures that have a concentration of API in the range of 500 mg/kg to 6200 mg/kg. In order to define the percentages of the excipients in the mixture, a mixture design was tailored on two factors (starch and lactose) at nine different levels (0%, 17%, 25%, 33%, 50%, 67%, 75%, 83%, and 100% for each excipient, the concentration of the other being the complement to 100%). Target dexamethasone concentrations were then randomly assigned to the various excipient ratios identified by the design.

In order to minimize the error associated with the weight of a very low mass of dexamethasone, two stock solid mixtures, one containing dexamethasone and starch (mixture A), and one constituted by dexamethasone and lactose (mixture B), at dexamethasone concentrations of 31,964 mg/kg and 32,395 mg/kg, respectively, were prepared and further diluted (by adding the proper amount of pure starch and lactose) in order to obtain the 27 analyzed mixtures. The masses of the diverse ingredients and the final dexamethasone concentrations in the mixtures are reported in Table 2.

Prior to the analyses, samples were inserted into glass vials and mixed to obtain a homogeneous distribution of the ingredients.

As previously mentioned, some tablets of Decadron^®^ (from different batches) were also analyzed. For this purpose, 10 tablets were individually ground, inserted into the vials (the same ones used for the analysis of the mixtures), and analyzed by NIR spectroscopy.

Dexamethasone (certified pharmaceutical secondary standard), starch, and α-lactose monohydrate (>99%) were obtained from Sigma-Aldrich KGaA (Darmstadt, Germany). Different batches of Decadron^®^ tablets 0.5 mg (European Pharmacopeia) were purchased in Italian municipal pharmacies.

Finally, in order to validate the model also predicting a real pharmaceutical formulation, tablets (coming from different lots) of a drug commercialized as Decadron were ground and analyzed.

### 4.2. Collection of FT-NIR Spectra

The spectra were acquired using a Nicolet 6700 FT-NIR instrument equipped with an integrating sphere (Thermo Scientific Inc., Madison, WI, USA), consisting of a tungsten-halogen source and an InGaAs detector. The analysis was performed by putting the vials containing the samples directly on the window of the integrating sphere. The analysis is therefore completely non-destructive, and the loss of sample is to be considered negligible (the sample is not altered during the measurement, and it can be recollected). Spectra were captured in the range of 4000 cm^−1^ to 10,000 cm^−1^ (nominal resolution of 4 cm^−1^). Two replicate spectra were collected on each mixture, using a different aliquot of the sample. In total, 54 signals were collected and exported by the Omnic software (Thermo Scientific Inc., Madison, WI, USA) to be processed in Matlab (R2015b; The Mathworks, Natick, MA, USA). 

### 4.3. Regression Strategies

Two different chemometric strategies have been applied in order to solve the regression problem finalized to the quantification of dexamethasone in mixtures and ground tablets; one consisting of the individual modeling of data preprocessed by different pretreatments (Strategy I), and one based on a multi-block method for ensemble preprocessing (Strategy II).

Applying Strategy I, data were pretreated by six different preprocessing approaches: standard normal variate (SNV) [48], first and second derivatives (D1 and D2, calculated by the Savitzky–Golay method, using 19-point windows and a third-degree polynomial) [49] and their combinations (SNV + D1 and SNV + D2). Therefore, six different regression models (one for each pretreated block) were built and solved by the Partial Least Squares (PLS) method (please refer to [31] for a description of the algorithm).

Strategy II contemplates the creation of regression models exploiting the sequential preprocessing through the orthogonalization (SPORT) [32] method, a multi-block approach derived from Sequential and Orthogonalized Partial Least Squares (SOPLS) [50]. SPORT allows the solving of a regression problem and, at the same time, the performing of ensemble preprocessing. Briefly, once the pretreatments to be tested have been defined, data are preprocessed and as many predictor blocks as tested pretreatments are obtained. Then, the model extracts information from all the blocks following a SOPLS-like algorithm. Very briefly, this means that the first preprocessed block (**X**_1_) is fitted to **Y** using PLS (this allows estimating scores **T**_X1_ and residuals **E**_Y1_). Then, the second block to be modeled (**X**_2_) is orthogonalized with respect to the scores obtained in the previous regression model (**T**_X1_). The orthogonalized **X**_2_ is then fitted to the residuals **E**_Y1_. All the following predictor blocks are then modeled following the same procedure, taking care to orthogonalize them with respect to the scores associated to all the other blocks previously modelled. This causes the information to be sequentially extracted from the various preprocessed blocks; for further details, the reader is addressed to {Formatting Citation}. Due to the fact that, in building sequential multi-block methods, the order of the input blocks can provide slightly diverse solutions, all the possible combinations of pretreated blocks were tested. The considered preprocessing approaches were the same mentioned above for Strategy I (bare mean-centering, SNV, D1, and D2).

### 4.4. Model Calibration and Validation

Disregarding the applied strategy, calibration models were built on training samples, whereas validation took place on test individuals. Ten test samples were selected by arranging the spectra in ascending order of API concentration and taking one sample out of every 5. A total of 44 individuals were then used for calibration. In all cases, replicate spectra from the same samples were kept in the same set. Validation samples were not used at any step of calibration model building.

In Strategy I, the optimal number of latent variables (LVs) to be extracted has been selected based on a 7-fold cross-validation procedure. Cross-validated models built extracting from 1 up to 10 LVs were calculated and then the optimal solution has been defined as the one which compromises between parsimony of LVs and minimization of the Root Mean Squares Error in Cross Validation (RMSECV). In strategy II, all the possible combinations of LVs (ranging from 0 to 10) among the blocks were tested, and then the optimal calibration model was defined by inspection of the RMSECV. Also in this case, the model achieving the lowest RMSECV involving a relatively small number of LVs was chosen as the optimal one.

## 5. Conclusions

The present work is a preliminary study to evaluate whether NIR spectroscopy can be used to quantify dexamethasone in mixtures with common excipients (starch and lactose). The aim of this work is to lay the foundations for the development of a rapid and non-destructive methodology for the detection of counterfeit or substandard dexamethasone-based formulations. For the realization of the regression models, a single-block (Strategy I) and a multi-block strategy (Strategy II) were applied and compared. The former gave acceptable results but was less accurate than the latter. Strategy II, based on the application of the SPORT method, provided more accurate and satisfying results, demonstrating its suitability for setting up a method for the quantification of dexamethasone in mixtures. Finally, this latter approach has been applied to quantify the inspected API in real formulations. In this case, the obtained results were satisfactory, but indicate that it is necessary to improve the model, probably by analyzing a greater number of mixtures and including other major excipients (for example, the dibasic calcium phosphate dihydrate) in order to obtain more accurate models on real formulations.

## Figures and Tables

**Table 1:** PLS models of the differently preprocessed spectra. Number of latent variables (LVs) extracted, and root mean square error in cross-validation (R^2^_CV_).

	Preprocessing	LVs	R^2^_CV_
Model I	Raw (+MC)	5	0.1023
Model II	SNV (+MC)	3	0.1990
Model III	D1 (+MC)	8	0.6275
Model IV	D2 (+MC)	8	0.7785
Model V	SNV + D1 (+MC)	9	0.6699
Model VI	SNV + D2 (+MC)	8	0.7457

**Table 2:** Quantity of the constituent components of the analyzed samples.

Sample	Starch (g)	Lactose (g)	Mixture A (g)	Mixture B (g)	Excipient Percentage		Dexamethasone Concentration (mg/kg)
					Starch	Lactose	
1	0.7404	2.2155	0.0113	0.0398	25	75	549
2	0.3953	0.9752	0.0494	0.0293	67	33	843
3	2.1843	0.7268	0.0699	0.0239	75	25	1001
4	0.4833	2.4065	0.0186	0.0968	17	83	1241
5	1.4298	1.4321	0.0712	0.0700	5	0.5	1513
6	0.4743	2.3706	0.0270	0.1340	17	83	1731
7	2.3552	0.4706	0.1453	0.0305	83	17	1876
8	0.9337	1.8688	0.0646	0.1301	33	67	2095
9	2.3817	0.4660	0.1771	0.0360	83	17	2230
10	0.9276	1.8312	0.0786	0.1556	33	67	2515
11	2.0627	0.6863	0.1902	0.0627	75	25	2693
12	0.4602	2.2760	0.0431	0.2236	17	83	2871
13	2.7125	0.0000	0.2897	0.0000	1	0	3084
14	2.2426	0.4459	0.2548	0.0495	83	17	3257
15	0.0000	2.6738	0.0000	0.3233	0	1	3495
16	1.7711	0.8855	0.2301	0.1138	67	33	3680
17	0.0000	2.6397	0.0000	0.3797	0	1	4074
18	0.8719	1.7464	0.1262	0.2572	33	67	4120
19	1.2987	1.3007	0.2006	0.1999	5	5	4296
20	0.6451	1.9355	0.1044	0.3149	25	75	4513
21	2.5627	0.0000	0.4382	0.0000	1	0	4668
22	1.6954	0.8476	0.3042	0.1541	67	33	4903
23	0.6337	1.8945	0.1194	0.3585	25	75	5133
24	2.4887	0.0000	0.5115	0.0000	1	0	5450
25	1.2385	1.2340	0.2655	0.2645	5	5	5680
26	0.0000	2.4418	0.0000	0.5579	0	1	6025
27	1.8178	0.6053	0.4344	0.1441	75	25	6181

## Data Availability

Data is contained within the article.

## References

[1] Pharmaceutical Security Institute (PSI) Pharma Crime: Geographic Distribution. https://www.psi-inc.org/geographic-distribution.

[2] Deepan T., Dhanaraju M.D. (2018). Stability indicating HPLC method for the simultaneous determination of dapagliflozin and saxagliptin in bulk and tablet dosage form. Curr. Issues Pharm. Med. Sci..

[3] Udhayavani S., Girija Sastry V., Govinda Rajan R., Ramya Krishna V., Tejaswi J.K.D. (2018). One step quantification analytical method and characterization of valsartan by LC-MS. Int. J. Appl. Pharm..

[4] Hussain R., Iqbal S., Shah M., Rehman W., Khan S., Rasheed L., Rahim F., Dera A.A., Kehili S., Elkaeed E.B. (2022). Synthesis of Novel Benzimidazole-Based Thiazole Derivatives as Multipotent Inhibitors of &alpha;-Amylase and &alpha;-Glucosidase: In Vitro Evaluation along with Molecular Docking Study. Molecules.

[5] Hussain R., Shah M., Iqbal S., Rehman W., Khan S., Rasheed L., Naz H., Al-Ghulikah H.A., Elkaeed E.B., Pashameah R.A. (2022). Molecular iodine-promoted oxidative cyclization for the synthesis of 1,3,4-thiadiazole-fused-[1,2,4]-thiadiazole incorporating 1,4-benzodioxine moiety as potent inhibitors of α-amylase and α-glucosidase: In vitro and in silico study. Front. Chem..

[6] Sadashivaiah R., Rohith G., Satheesha Babu B.K. (2019). Quantification of ropinirole hydrochloride in api and tablets by novel stability-indicating rp-hplc method: It’s validation and forced degradation studies. Int. J. Appl. Pharm..

[7] Habash I.W., Al-Shdefat R.I., Hailat M.M., Dayyih W.A. (2020). A stability indicating rp-hplc method development for simultaneous estimation of alogliptin, pioglitazone, and metformin in pharmaceutical formulations. Acta Pol. Pharm. -Drug Res..

[8] Bulduk İ., Aydın B.S. (2020). Simple high-performance liquid chromatographic method for determination of donepezil hcl in pharmaceutical formulations. J. Chem. Metrol..

[9] Tomilova E., Kurgachev D., Kulagina D., Sysolyatin S., Krylova S., Novikov D. (2021). Development of HPLC-Method for Simultaneous Determination of API and Related Components in Thiowurtzine: A New Non-Narcotic Analgesic. Chromatographia.

[10] Zhong L., Gao L., Li L., Nei L., Wei Y., Zhang K., Zhang H., Yin W., Xu D., Zang H. (2022). Method development and validation of a near-infrared spectroscopic method for in-line API quantification during fluidized bed granulation. Spectrochim. Acta -Part A Mol. Biomol. Spectrosc..

[11] Ali S.L. (2000). Counterfeit drugs and analytical tools for their discrimination: European perspectives. Pharm. Chem. J..

[12] Olsen B.A., Kiehl D.E. (2006). Authentication and fingerprinting of suspect counterfeit drugs. Am. Pharm. Rev..

[13] Blackstone E.A., Fuhr J.P., Pociask S. (2014). The health and economic effects of counterfeit drugs. Am. Health Drug Benefits.

[14] Sarraguça M.C., Lopes J.A. (2009). Quality control of pharmaceuticals with NIR: From lab to process line. Vib. Spectrosc..

[15] Moffat A.C., Assi S., Watt R.A. (2010). Identifying counterfeit medicines using near infrared spectroscopy. J. Near Infrared Spectrosc..

[16] Järvinen K., Hoehe W., Järvinen M., Poutiainen S., Juuti M., Borchert S. (2013). In-line monitoring of the drug content of powder mixtures and tablets by near-infrared spectroscopy during the continuous direct compression tableting process. Eur. J. Pharm. Sci..

[17] Makraduli L., Makreski P., Goracinova K., Stefov S., Anevska M., Geskovski N. (2020). A Comparative Approach to Screen the Capability of Raman and Infrared (Mid- and Near-) Spectroscopy for Quantification of Low-Active Pharmaceutical Ingredient Content Solid Dosage Forms: The Case of Alprazolam. Appl. Spectrosc..

[18] Roggo Y., Chalus P., Maurer L., Lema-Martinez C., Edmond A., Jent N. (2007). A review of near infrared spectroscopy and chemometrics in pharmaceutical technologies. J. Pharm. Biomed. Anal..

[19] Gerich A., Verhoog J., Damen M., Verhoeven W., Chamarthy S.P., Besseling R. (2017). Detection of Lumps in Powder Blends by Inline NIR. Pharm. Technol..

[20] Storme-Paris I., Rebiere H., Matoga M., Civade C., Bonnet P.-A., Tissier M.H., Chaminade P. (2010). Challenging Near InfraRed Spectroscopy discriminating ability for counterfeit pharmaceuticals detection. Anal. Chim. Acta.

[21] Rodionova O., Pomerantsev A., Houmøller L., Shpak A., Shpigun O. (2010). Noninvasive detection of counterfeited ampoules of dexamethasone using NIR with confirmation by HPLC-DAD-MS and CE-UV methods. Anal. Bioanal. Chem..

[22] Henrique Frasson Scafi S., Pasquini C. (2001). Identification of counterfeit drugs using near-infrared spectroscopy. Analyst.

[23] Horby P., Lim W.S., Emberson J.R., Mafham M., Bell J.L., Linsell L., Phil D., Staplin N., Brightling C., Med F. (2021). Dexamethasone in Hospitalized Patients with COVID-19. N. Engl. J. Med..

[24] Fahriani M., Ilmawan M., Fajar J.K., Maliga H.A., Frediansyah A., Masyeni S., Yusuf H., Nainu F., Rosiello F., Sirinam S. (2021). Persistence of long COVID symptoms in COVID-19 survivors worldwide and its potential pathogenesis—A systematic review and meta-analysis. Narra J..

[25] Fajar J.K., Ilmawan M., Mamada S., Mutiawati E., Husnah M., Yusuf H., Nainu F., Sirinam S., Keam S., Ophinni Y. (2021). Global prevalence of persistent neuromuscular symptoms and the possible pathomechanisms in COVID-19 recovered individuals: A systematic review and meta-analysis. Narra J..

[26] Miyabo S., Nakamura T., Kuwazima S., Kishida S. (1981). A comparison of the bioavailability and potency of dexamethasone phosphate and sulphate in man. Eur. J. Clin. Pharmacol..

[27] Arthur K.E., Wolff J.-C., Carrier D.J. (2004). Analysis of betamethasone, dexamethasone and related compounds by liquid chromatography/electrospray mass spectrometry. Rapid Commun. Mass Spectrom..

[28] Vakili H., Wickström H., Desai D., Preis M., Sandler N. (2017). Application of a handheld NIR spectrometer in prediction of drug content in inkjet printed orodispersible formulations containing prednisolone and levothyroxine. Int. J. Pharm..

[29] Deeley C.M., Spragg R.A., Threlfall T.L. (1991). A comparison of Fourier transform infrared and near-infrared Fourier transform Raman spectroscopy for quantitative measurements: An application in polymorphism. Spectrochim. Acta Part A Mol. Spectrosc..

[30] Wold S., Martens H., Wold H., Kågström B., Ruhe A. (1983). The Multivariate Calibration Problem in Chemistry Solved by the PLS Method. Matrix Pencils. Lecture Notes in Mathematics.

[31] Martens H., Naes T. (1989). Multivariate Calibration.

[32] Roger J.-M., Biancolillo A., Marini F. (2020). Sequential preprocessing through ORThogonalization (SPORT) and its application to near infrared spectroscopy. Chemom. Intell. Lab. Syst..

[33] Geladi P., Kowalski B.R. (1986). Partial least-squares regression: A tutorial. Anal. Chim. Acta.

[34] Biancolillo A., Marini F., Ruckebusch C., Vitale R. (2020). Chemometric strategies for spectroscopy-based food authentication. Appl. Sci..

[35] Maraphum K., Saengprachatanarug K., Wongpichet S., Phuphuphud A., Posom J. (2022). Achieving robustness across different ages and cultivars for an NIRS-PLSR model of fresh cassava root starch and dry matter content. Comput. Electron. Agric..

[36] Mishra P., Marini F., Biancolillo A., Roger J.-M. (2021). Improved prediction of fuel properties with near-infrared spectroscopy using a complementary sequential fusion of scatter correction techniques. Talanta.

[37] Martínez L., Peinado A., Liesum L. (2013). In-line quantification of two active ingredients in a batch blending process by near-infrared spectroscopy: Influence of physical presentation of the sample. Int. J. Pharm..

[38] De Leersnyder F., Peeters E., Djalabi H., Vanhoorne V., Van Snick B., Hong K., Hammond S., Liu A.Y., Ziemons E., Vervaet C. (2018). Development and validation of an in-line NIR spectroscopic method for continuous blend potency determination in the feed frame of a tablet press. J. Pharm. Biomed. Anal..

[39] Foschi M., Marziale M., Biancolillo A. (2022). Advanced Analytical Approach Based on Combination of FT-IR and Chemometrics for Quality Control of Pharmaceutical Preparations. Pharmaceuticals.

[40] Wright S. (1921). Correlation and Causation. J. Agric. Res..

[41] Biancolillo A. (2016). Method Development in the Area of Multi-Block Analysis Focused on Food Analysis. Ph. D Thesis.

[42] Biancolillo A., Næs T., Cocchi M. (2019). The Sequential and Orthogonalized PLS Regression for Multiblock Regression: Theory, Examples, and Extensions. Data Fusion Methodology and Applications.

[43] Biancolillo A., Marini F., Roger J.-M. (2020). SO-CovSel: A novel method for variable selection in a multiblock framework. J. Chemom..

[44] Wold S., Johansson E., Cocchi M. (1993). PLS—Partial Least-Squares Projections to Latent Structures. 3D QSAR Drug Design.

[45] Cocchi M., Biancolillo A., Marini F., Jaumot J., Bedia C., Tauler R. (2018). Chemometric Methods for Classification and Feature Selection. Data Analysis for Omic Sciences: Methods and Applications, Comprehensive Analytical Chemistry.

[46] Foo W.C., Widjaja E., Khong Y.M., Gokhale R., Chan S.Y. (2018). Application of miniaturized near-infrared spectroscopy for quality control of extemporaneous orodispersible films. J. Pharm. Biomed. Anal..

[47] Cournoyer A., Simard J.-S., Cartilier L., Abatzoglou N. (2008). Quality control of multi-component, intact pharmaceutical tablets with three different near-infrared apparatuses. Pharm. Dev. Technol..

[48] Barnes R.J., Dhanoa M.S., Lister S.J. (1989). Standard normal variate transformation and de-trending of near-infrared diffuse reflectance spectra. Appl. Spectrosc..

[49] Savitzky A., Golay M.J.E. (1964). Smoothing and differentiation of data by simplified least squares procedures. Anal. Chem..

[50] Næs T., Tomic O., Mevik B.-H., Martens H. (2011). Path modelling by sequential PLS regression. J. Chemom..

